# Protective effects of arecanut seed phenols in retinoic acid induced osteoporosis and the potential mechanisms explored by network pharmacology

**DOI:** 10.3389/fendo.2024.1472146

**Published:** 2024-10-10

**Authors:** Min-min Tang, Li-ping Sun, Fei Song, Hua Chen

**Affiliations:** ^1^ Coconut Research Institute, Chinese Academy of Tropical Agricultural Sciences, Wenchang, Hainan, China; ^2^ Hainan Betel Nut Engineering Technology Research Center, Wenchang, Hainan, China; ^3^ Faculty of Food Science and Engineering, Kunming University of Science and Technology, Kunming, Yunnan, China

**Keywords:** arecanut seed, phenols, osteoporosis, network pharmacology, molecular docking

## Abstract

**Background:**

Arecanut seed is an important traditional medicine in Southeast Asia. It has been presented in a clinical formula to treat osteoporosis (OP) in China. Arecanut seed is abundant in phenols. However, most of current studies mainly focused on estrogen-deficient osteoporosis (OP) model of arecanut seed phenols (ASP), there is still a lack of roundly research about molecular mechanism of ASP therapy on OP and its influence on in drug-induced bone loss.

**Materials and methods:**

To explore potential molecular mechanisms and the effects of ASP on OP, network pharmacology, molecular docking methods and a retinoic acid-induced OP rat model were employed in this study. According to the network pharmacology method, OP related targets and ASP compound related targets were collected from databases to obtain hub targets and top active chemicals in ASP treating OP. The potential therapic pathways were also calculated. Binding capacities of top active chemicals to hub targets were analyzed by molecular dock assay. In the animal experiment, osteocalcin (OCN) levels and alkaline phosphatase (ALP) activity in serum of all the rats were determined. The views of bone section were stained to observe the bone micro-structure of ASP affects. Bone mineral density (BMD), cortical bone thickness (CBT), area ratio of bone cortex (CAR) and area ratio of bone trabecula (TAR) were obtained from micro computed tomography to evaluate the effectiveness of ASP on bone loss.

**Conclusion:**

Three hub genes and three top active compounds were screened by network pharmacology analysis and they combined well with each other. ASP had positive effects on alleviating RA-induced bone loss by regulating the expression of the hub genes. Signals in IL-17 pathway were predicted and primarily verified being potential targets in ASP treating OP.

## 1 Introduction

The seed of ripe arecanut fruit (*Areca catechu* L.) has been widely used for medicinal purposes in South Asia from ancient. In China, it is a famous traditional Chinese medicine (TCM) and has been recorded in the Chinese Pharmacopoeia from 1959. Arecanut seed is one of the clinically used common drugs for the treatment of gastrointestinal, edematous, and parasitic diseases (1). Modern pharmacological studies demonstrated that the seed displayed excellently in antioxidant, antidiabetics, antihypotensive (2, 3), anti-depressant (4), anti-inflammatory (5, 6), analgesic (6), skin-whitening effects (7), and can regulate bone metabolism in ovariectomized (OVX)-induced OP rats (8, 9). Those bioactivities mainly attributed to the facts that the seed supplies various functional constituents such as phenols (2, 3, 8), alkaloids (10), polysaccharides (11), fibers and fatty acids. Among the detected ingredients, phenols can be considered one group of the most important secondary metabolites in arecanut seed as they take more than 30% (w/w) of the dry material and they have exhibited a spectrum of pharmacological activities (1, 12).

Osteoporosis (OP) is a systemic skeletal disease that is primarily characterized by the impairment of bone mineral density (BMD) and microarchitecture with a consequent increase in bone fragility and a susceptibility to fractures. By the pathogenesis, OP could be divided into primary and secondary OP. The primary OP, also called degenerative OP, is mainly caused by aging and endocrine environment (13). The secondary OP can be caused by a variety of factors such as genetics, drugs (14), and other diseases. Currently, the prevention and treatment strategies for the two types of OP are mainly comprised bone resorption inhibitors, production promoters, and bone minerals, such as calcitonin, bisphosphonates, fluorides, etc. Generally, in research, aging or estrogen-inefficient OP models were chosen for research of the primary OP *in vivo*, while drug-indued OP models were selected for the secondary. Corresponding to the related molecular levels, the classical signal transduction pathways including the receptor activator of nuclear factor-κB (RANK)/RANK ligand (RANKL)/osteoprotegerin (OPG) axis, wingless (Wnt) pathway, and the nuclear factor kappa-B (NF-κB) signaling pathways have been considered as the important therapeutic targets in the treatment of OP.

As a potential alternative for treatment of various diseases, the therapeutic actions of TCM are characterized following the “multi-component, multi-pathway and multi-target” synergism, and precisely because of this, it is difficult to accurately study the mechanism actions of them in the treatment process. Adaptively, an innovative pharmacological methodology called network pharmacology was firstly suggested in 2007 by Andrew L Hopkins (15). Network pharmacology explores the relationship among drug components and disease targets by revealing the role and treatment mechanism of the aiming materials on diseases. The results of this methodology can elucidate the interactions between drugs and targets of disease through an unambiguous network of interactions. Subsequently, molecular docking, an important tool of the drug discovery process, can be used to simulate the interactions between small molecule ligands (drugs) and biomacromolecule receptors (target proteins). The component could calculate binding affinity and acting sites between a drug and a target protein. There have been several research about employing network pharmacology and molecular docking analysis combined with validation of experiments *in vitro* or *in vivo* to explore the mechanisms of TCM on OP therapy (16–18).

Retinoic acid has been clinically applied in a variety of dermatological conditions such as skin cancer, psoriasis, acne, and ichthyosis, but there is obvious damage on bone metabolism in case of exceed use (19). Thus, RA is popularly used to establish a drug-induced OP model in animals as its procedure is easy to preform and takes a short time. The RA-induced OP can avoid stress reaction and the symptoms is similar to human OP, especially the view of bone histomorphology. It is noted that arecanut seed phenols (ASP) can ameliorate bone metabolism in ovariectomized (VOX)-rats with osteoporosis by inhibiting Wnt/β-catenin pathway (8). However, there is still a lack of a roundly effectiveness evaluation of ASP on bone loss in secondary OP. Additionally, the demonstrated molecular mechanisms of ASP on OP were some classical pathways in current studies, lack of extent signaling investigation. Thus, this study was aimed to investigate the molecular mechanisms of ASP in treating secondary OP by network pharmacology and verified the predicting results in RA-induced OP in rat.

## 2 Materials and methods

### 2.1 Plant materials and chemical reagents

The ASP was prepared as previously reported (2). Tablets of alendronate sodium was bought from Merck & Co., Inc. (Hangzhou, China) and retinoic acid (purity ≥ 99%) was purchased from Shanghai yuanye Bio-Technology Co., Ltd (Shanghai, China). Determination of serum calcium, phosphorus, alkaline phosphatase (ALP), and osteocalcin (OCN) were performed with the corresponding assay kits purchased from Nanjing Jiancheng Bioengineering Institute (Nanjing, China). Serum tartrate-resistant acid phosphatase (TRAP) activity kit was obtained from Beyotime Biotechnology (Shanghai, China) and TRAP staining kit was purchased from Sigma-Aldrich. Inc. (Missouri, USA). Trizol reagent and fast SYBR-green reagent were obtained from Thermo Fisher Scientific (Invitrogen, Carlsbadd, USA). All the normal reagents, such as chloral hydrate, hydroquinone, and paraformaldehyde, used in the experiment were analytically pure.

### 2.2 Network pharmacology

#### 2.2.1 Prediction and screening of compounds-OP related targets

In this section, forty-seven phenols were collected for network pharmacology analysis, including forty phenols detected in previous study (2) and other seven obtained from the pharmacological database of Traditional Chinese Medicine Systems Pharmacology Database and Analysis Platform (TCMSP, http://lsp.nwu.edu.cn/tcmsp.php) (20) with the keyword of “*Arecae semen*”. Those compounds were screened by two key absorption, distribution, metabolism and excretion (ADME) parameters of oral bio-availability (OB ≥ 30%) and drug similarity (DL ≥ 0.18) (21). The selected phenolic compounds were picked up as the potential candidates and their related targets of *Rattus norvegicus* were predicted on the TCMSP and Swiss TargetPrediction platform (www.swisstargetprediction.ch, updated on 18^th^ February 2024) (22). Then all the targets were annotated to the corresponding genes via the Uniprot platform (https://www.uniprot.org/) (23). OP-related targets were retrieved by searching the term “osteoporosis” from two public data-bases of GeneCards (https://www.genecards.org/) and OMIM (https://omim.org/), which are two most comprehensive banks about diseases targets, to get OP-related targets. After deleting the repetitive targets, the rest ones were transformed to the corresponding proteins verified in *Rattus norvegicus*.

Subsequently, we applied Veeny 2.1 to draw a venn diagram for the intersectional targets of the both to identify the candidate target genes of arecanut seed phenols against OP. We constructed a drug-component-disease target network using Cytoscape 3.9.1 software, and calculated the network parameters using the Network Analyzer tool to find key components of OP. The obtained intersection targets were imported into the String database (https://string-db.org/) for PPI network information. We used Cytoscape 3.9.1 software to visualize the network and filter out the key targets. Finally, we imported the targets screened from the PPI network into the Metascape database (https://metascape.org/gp/index.html) for GO and KEGG enrichment analysis (minimum overlap=3, *p* value cutoff=0.01 and minimum enrichment=1.5). The results were presented as bar graphs using the *R* programming language software (version 3.6.3) for bioinformatics-related data analysis.

#### 2.2.2 Molecular docking

Based on the results of network pharmacology analysis, three top key compounds and three core target proteins were selected as the ligands and receptors in docking verification. The structures of the ligands were obtained from the Pubchem database (https://pubchem.ncbi.nlm.nih.gov/) and the three-dimensional structures of the target proteins were obtained from the RCSB PDB database (https://www.rcsb.org/). Then, the binding affinity of the ligand with the receptor was evaluated by calculating the binding energy by autodock vina software.

### 2.3 *In vivo* animal experiment verification

All animal experimental procedures were approved by the Institutional Animal Care and Use Committee (IACUC) of Kunming University of Science and Technology. Generally, a total of thirty-two Sprague-Dawley (SD) female rats (8 weeks, body weight ranging in 140~180g) were purchased from the Laboratory Animal Center of Kunming Medical University (Kunming, China). The rats were raised in the same condition maintained at the temperature of 20~26 °C, the relative humidity of 40~70%, and the air cleanliness of grade seven. The standard pellet feed and water ad libitum were given with a 12 h light-dark cycle. After acclimating for a week, eight rats were randomly selected as the normal control (NC) group and they were subsisted with normal diet until the end of the experiment.

Starting from now, the rest twenty-four rats were feed with normal diet along with intragastric administration of retinoic acid at 75 mg/kg body weight per day for two weeks. In addition, the NC group rats received normal saline until the experiment ended. Thereafter, the rats fed with retinoic acid were randomly divided into three groups, labelled as model control (MC) group, positive control (PC) group and ASP group. The rats in those three groups orally received normal saline, 5 mg/kg bw alendronate and 100 mg/kg bw ASP for three weeks, respectively. The bw of each rat was recorded to adjust the oral dose weekly until the experiment ended.

At the end of the experiment, all the rats were sacrificed after an overnight fast. Blood sample of each rat was collected from the ventral aorta. Serum was obtained via centrifugating the blood sample at 3500 r/min for 15 min at 4 °C and stored in -80 °C for pending experiments. Femurs and tibias, removing the adhered fascia and meat, were washed with normal saline for the following determination.

#### 2.3.1 Measurements of serum biochemical parameters

The levels of serum calcium, phosphorus, ALP, TRAP, and OCN, were tested using the corresponding commercial kits. All experimental procedures were operated strictly in accordance with the kit instructions.

#### 2.3.2 Quantitative analysis of bone quality

Bone quality indices were analyzed with femurs and tibias. The length and diameter of the right femurs and tibias was measured with a vernier caliper and recorded. Bone calcium and phosphorus contents of the right tibia were determined by a flame atomic absorption spectrometer (novAAfi 350, Analytikjena, Germany) (24).

Bone mineral density (BMD), cortical bone thickness (CBT), area ratio of bone cortex (CAR) and area ratio of bone trabecula (TAR) of the left femurs were obtained by a quantitative computed tomography assay with a micro-computed tomography (micro-CT) scanner (Latheta LCT-200, Hitachi Aloka Medical, Japan) (25). The scans were used an X-ray tube potential of 70 kV, intensity of 80 μA and isotropic voxel size of 9.0 μm.

The micro-CT scanning software was used to analyze the images to get the results.

#### 2.3.3 Histomorphological analysis

As the micro-CT scanning finished, the left femurs were rapidly collected and immediately dipped in 4% paraformaldehyde solution for 24 h. After the decalcification, standard sampling and pruning procedures, the tissues were embedded in paraffin. The paraffin-embedded tibias were cut into 2~3 µm-thick sections transversely at the epiphyseal end and longitudinally at the shaft, respectively. Then the dissected sections were stained with hematoxylin and eosin (H&E). The proximal tibias were embedded in paraffin, sectioned, stained with TRAP. All the stained sections were observed on a DP80 Digital Camera System (Olympus, Tokyo, Japan).

#### 2.3.4 Determination of gene contents in femurs by reverse transcription polymerase chain reaction

We chose a total of top 3 targets with high degree value to validate according to the “degree value” of network pharmacology. The total RNA of left femurs from the NC, MC, PC and ASP groups (n=3) was extracted as follows. After crushing the tibias with a bone forceps, the tissue pieces were put it into a dried mortar to grind into fine power with liquid nitrogen. Then total RNA was extracted with Trizol reagent according to the instructions of the manufacturer. The purity and integrity of the RNA extracts were ensured by a micro-spectrophotometer (NanoDrop One, Thermoscientific, Carlsbadd, USA). Then, the cDNAs were prepared by inversely transcribing with high-capacity PrimeScript RT reagent Kit (Takara, Tokyo, Japan). RT-qPCR was performed with an ABI 96 qPCR System (QuantStudio 3, Waltham, Massachusetts, USA) using the fast SYBR-green reagent as the fluorescence dye. Gene-specific primers used for real-time reactions were obtained from Sangon Biotech and shown in **Supplementary Table S1**. The total reaction volume of the realtime PCR was 20 μL and the reaction condition was set following previously performed (26). The gene expression was calculated according to the 2*^−△△^* ^CT^ method.

### 2.4 Statistical analyses

Data were expressed as mean ± standard deviation. The data were analyzed by one-way ANOVA and Tukey’s procedure was used to determine the significant differences (*p*<0.05). All analysis was performed on the data using SPSS software package (version 19.0, IBM Inc., Chicago, IL, USA).

## 3 Results

### 3.1 Anti-OP efficacy of OP through network pharmacology

A total of 25 bioactive phenols from ASP were screened out and the detailed information of them is described in **Supplementary Table S2**. Based on the Swiss Target Prediction platform, a total of 238 compound-related targets of rat were identified. Taking “osteoporosis” as the search term, OP-related targets were obtained from the GeneCards an OMIM. After merging and removing duplicate targets, a total of 1455 targets related to OP in rat were reserved for the following analysis.

**Figure 1:**
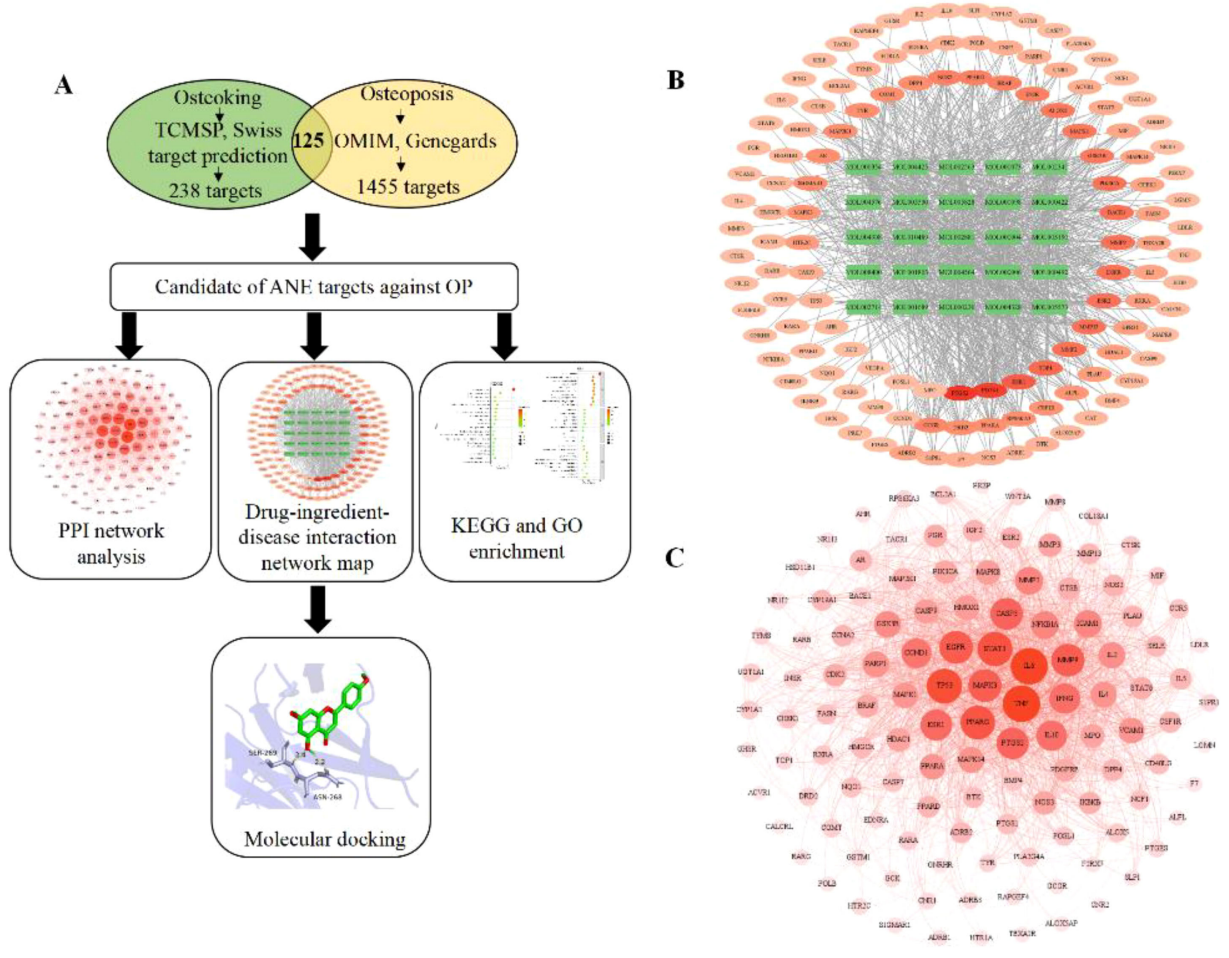
Network pharmacology predicts mechanisms and targets of ASP therapy for OP. **(A)** Network pharmacology procedures. **(B)** Interaction network between ASP compounds and potential targets (The green rectangle is active compounds of ASP; The red to light red oval is the intersection of target disease and ASP). **(C)** Construction of a PPI network of all overlapped potential target proteins (The darker the color, the larger node, indicating that the node plays a central role in the PPI network).

The Venn diagram (**Figure 1A**) showed that 125 targets were at the intersection of compound targets and disease targets, which were also predicted as the potential targets of ASP for alleviating OP. The interaction network between ASP compounds and potential targets (**Figure 1B**) includes 150 nodes and 854 edges. After network topology algorithm analysis, some potential key components for OP therapy with ASP were identified, among which kaempferol, isorhamnetin, and acacetin are ranked the top three with the largest size of nodes (**Supplementary Table S3**). The 125 potential targets were then subjected to the construction of a PPI network composited by 122 nodes and 1609 edges (**Figure 1C**). The interaction relationship among ASP and target proteins were calculated, with the top 3 highest protein nodes being filtered by the size of degree (**Supplementary Figure S1**). The filtered targets were tumor necrosis factor (TNF-α, n=85), interleukin-6 (IL-6, n=84), and cellular tumor protein p53 (TP53, n=78).

**Figure 2:**
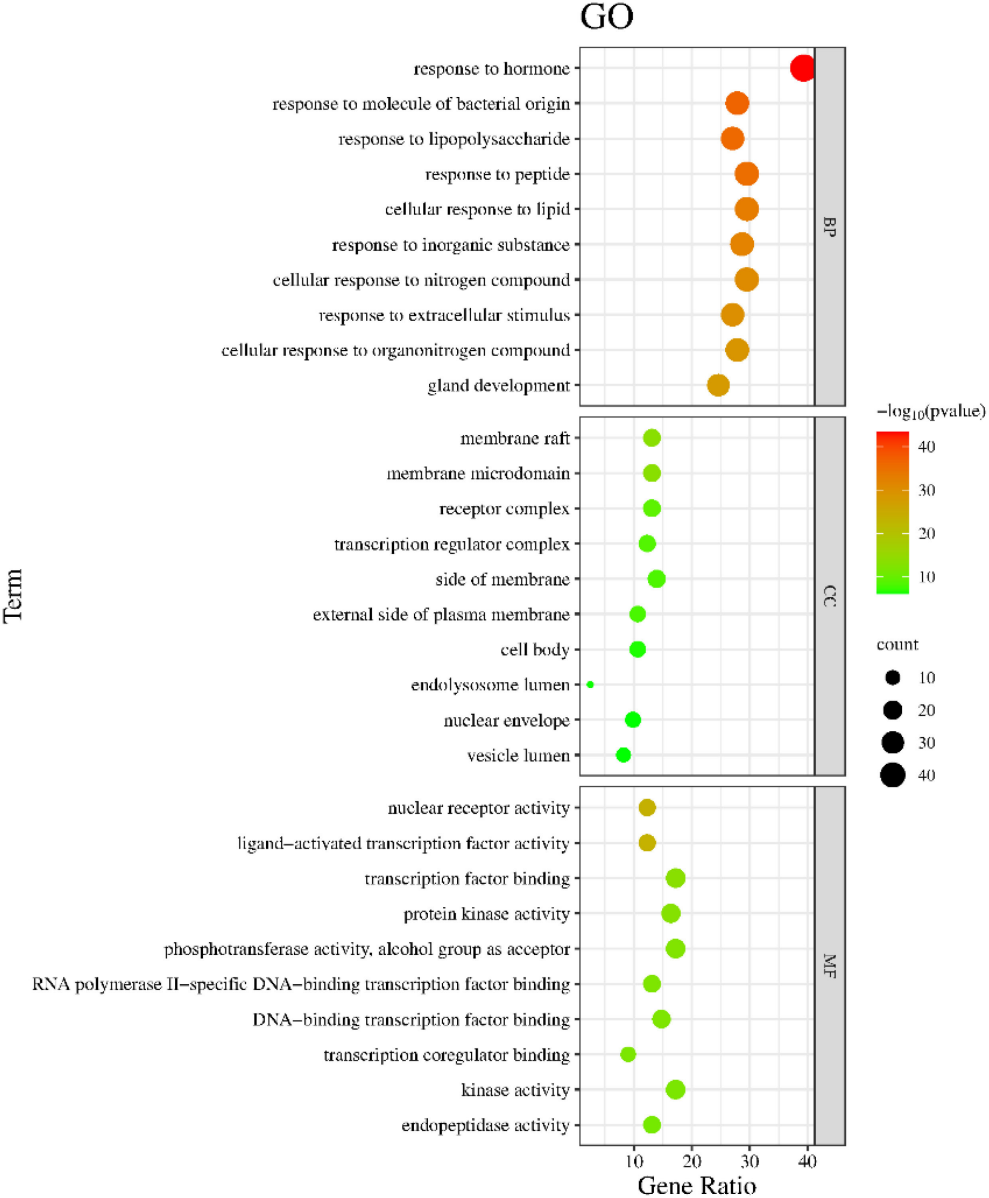
The GO analysis of interacting targets.

**Figure 3:**
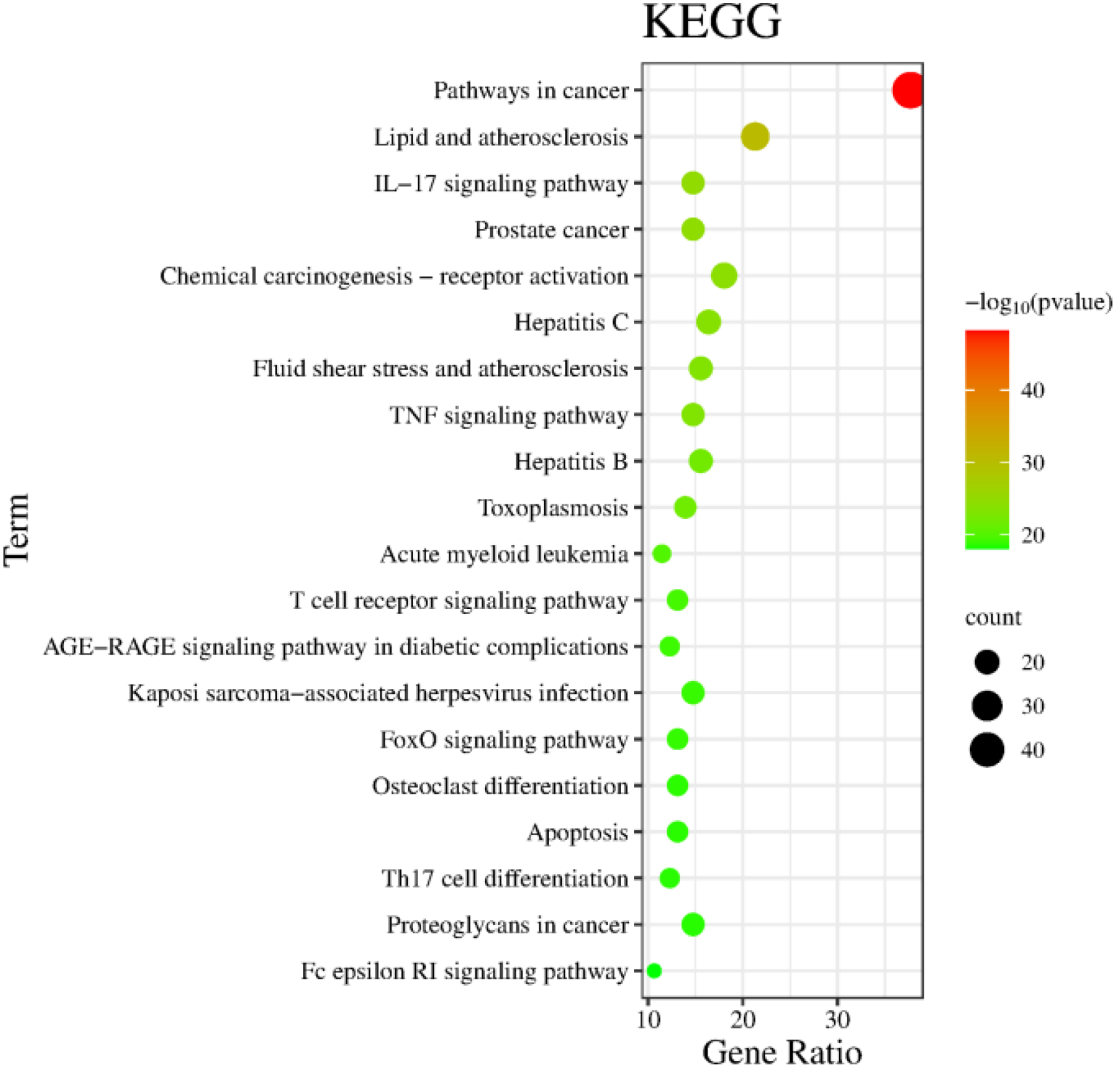
The KEGG analysis of interacting targets.

The Metascape database were used for GO enrichment analysis with the 125 target genes. As a result, 1753 GO items were obtained, including 1571 biological processes (BP), 58 cellular components (CC) and 124 molecular functions (MF). According to the *p* value and the number of enriched genes, the top 10 enriched BPs, CCs and MFs were selected for generating a visualized bubble diagram (**Figure 2**). According to KEGG pathway enrichment analysis, a total of 185 signaling pathways were screened and the top 20 ones were selected to draw the KEGG functional enrichment bubble diagram. Pathways in cancer, lipid and atherosclerosis, and Interleukin-17 (IL-17) signaling pathway were the three top ones (**Figure 3**).

**Figure 4:**
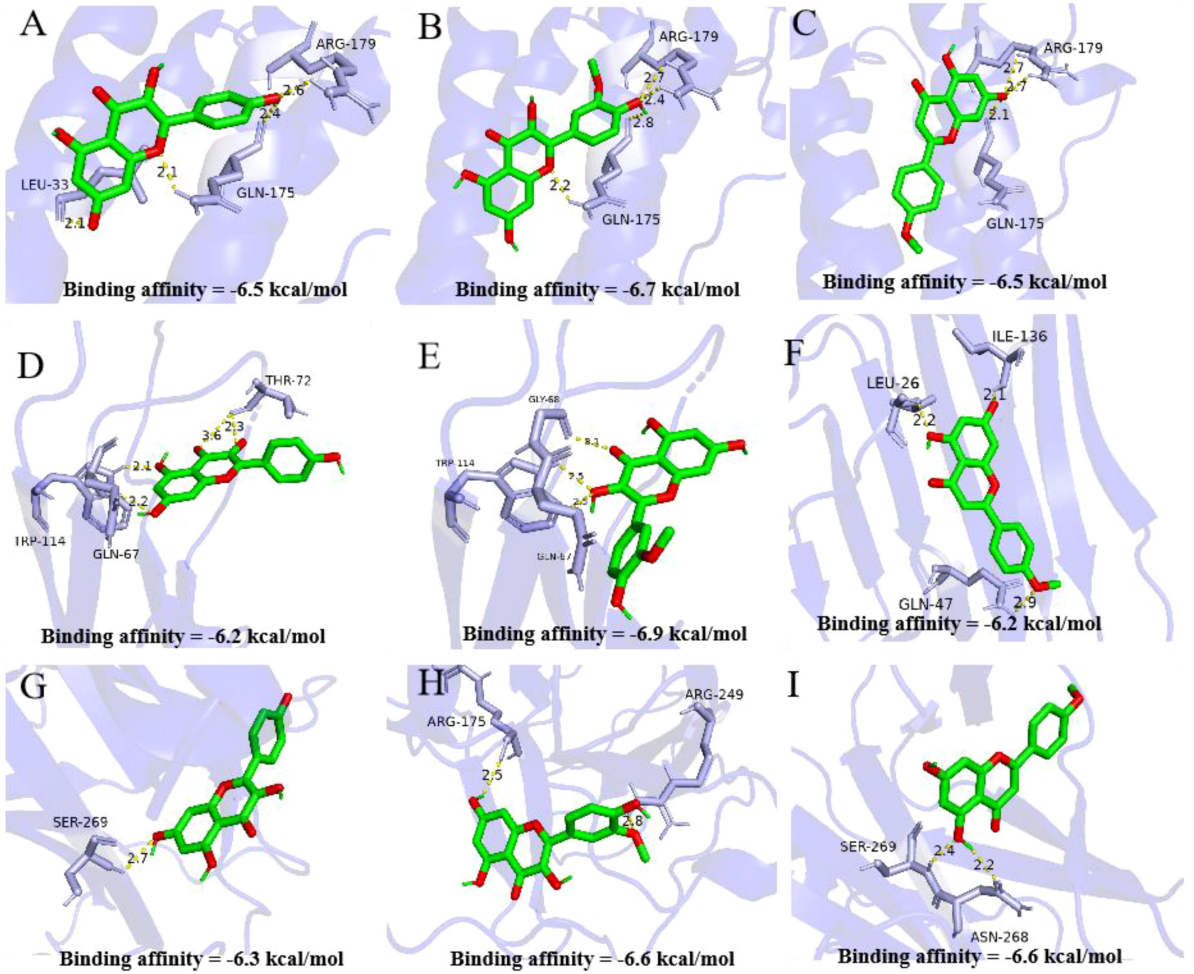
Molecular docking analysis of active compounds and core targets. **(A-C)** Kaempferol-TNF-α, Isorhamnetin-TNF-α, Acacetin-TNF-α; **(D-F)** Kaempferol-IL-6, Isorhamnetin IL-6, Acacetin IL-6; **(G-I)** Kaempferol-TP53, Isorhamnetin-TP53, Acacetin-TP53. Note: Data are mean ± SD, different lowercases mean there is significant difference between the corresponding groups at *p<0.05*. Lowercases have the same meaning in the following figures.

In this part, we used the molecular docking stimulation to investigate the binding capacities between the bioactive components of ASP and the predicted targets. The binding affinity reflects the potential of binding between the two molecules. The lower the binding energy, the higher the affinity of the receptor and the ligand, the more stable the conformation (27). According to **Figure 4**, all the bioactive components of ASP demonstrated good binding abilities with the receptors, suggesting that the ASP had a strong tendency as a therapeutic strategy for OP via these targets, including TNF-α, IL-6 and TP53. The results showed that isorhamnetin displayed the strongest binding ability with TNF-α (docking score=-6.9), IL-6 (docking score=-6.7), and TP53 (docking score=-6.6). Acacetin has a considerable binding ability to TP53 (docking score=-6.6).

### 3.2 Animal experiment verification ASP improves in osteoporotic rats

#### 3.2.1 The effects of ASP on serum levels of bone turnover markers

**Figure 5:**
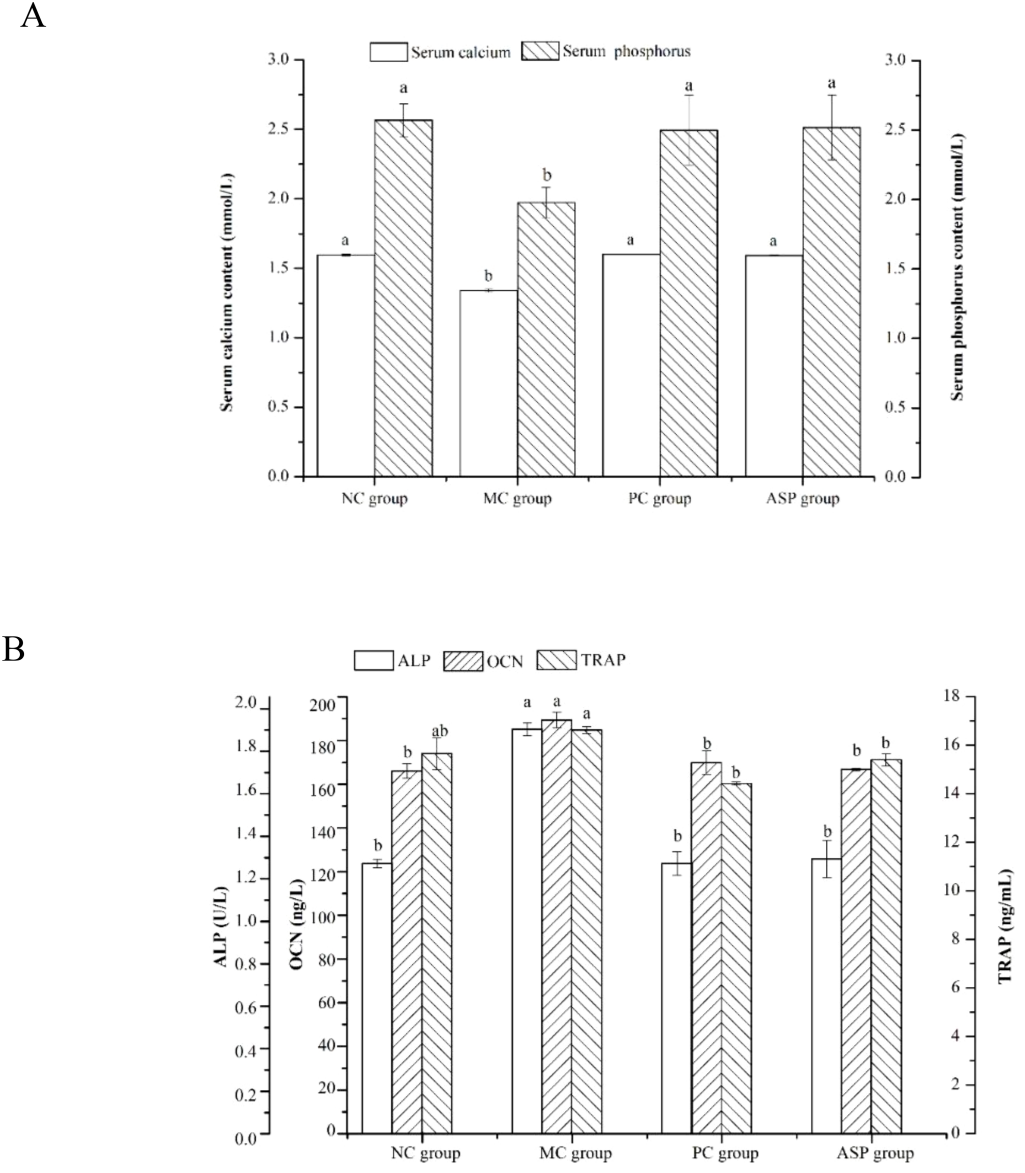
Effects of ASP on serum parameters in retinoic acid-induced osteoporosis rats. **(A)** Effects of ASP on serum calcium and phosphorus contents in retinoic acid-induced osteoporosis rats. **(B)** Effects of ASP on serum OCN content and activities of ALP and TRAP in retinoic acid-induced osteoporosis rats. Different lowercases mean there is significant difference between the corresponding groups at p<0.05. Lowercases have the same meaning in the following figures.

Concentrations of some bone turnover markers in serum, such as calcium, phosphorus, ALP, OCN and TRAP, can reflect the essence of bone metabolism, as they exhibit the levels bone formation and bone resorption directly or indirectly in mammals (28). In this study, the effects of ASP on these parameters in RA-induced OP rats were analyzed. As shown in **Figure 5A**, the serum calcium and phosphorus contents in the MC group significantly decreased (*p<0.05*) when compared with those of the NC group. The calcium and phosphorus contents in the PC and ASP groups significantly increased than those in the MC group *(p<0.05*). There were no significant differences of the two parameters between the ASP and NC groups (*p>0.05*). The levels of ALP and OCN were considerably increased in the MC group (*p<0.05*; **Figure 5B**) comparing with the NC group. Compared with those in the MC group, the levels of ALP and OCN the ASP group were significantly increased by 32.02% and 11.95%, respectively. In addition, there were no significant differences of those two biochemical indicators compared with the PC group and the NC group, respectively (*p>0.05*). However, it was a pity that there was no significant improvement on TRAP of ASP observed (*p>0.05*).

The levels of ALP and OCN were considerably increased in the MC group (*p<0.05*; **Figure 5B**) compared with the NC group. Compared with those in the MC group, the levels of ALP and OCN the ASP group were significantly increased by 32.02% and 11.95%, respectively. In addition, there were no significant differences of those two biochemical indicators compared with the PC group and the NC group, respectively (*p>0.05*). However, it was a pity that there was no significant improvement on TRAP of ASP observed (*p>0.05*).

#### 3.2.2 The effects of ASP on biochemical and biomechanical parameters of bone

**Figure 6:**
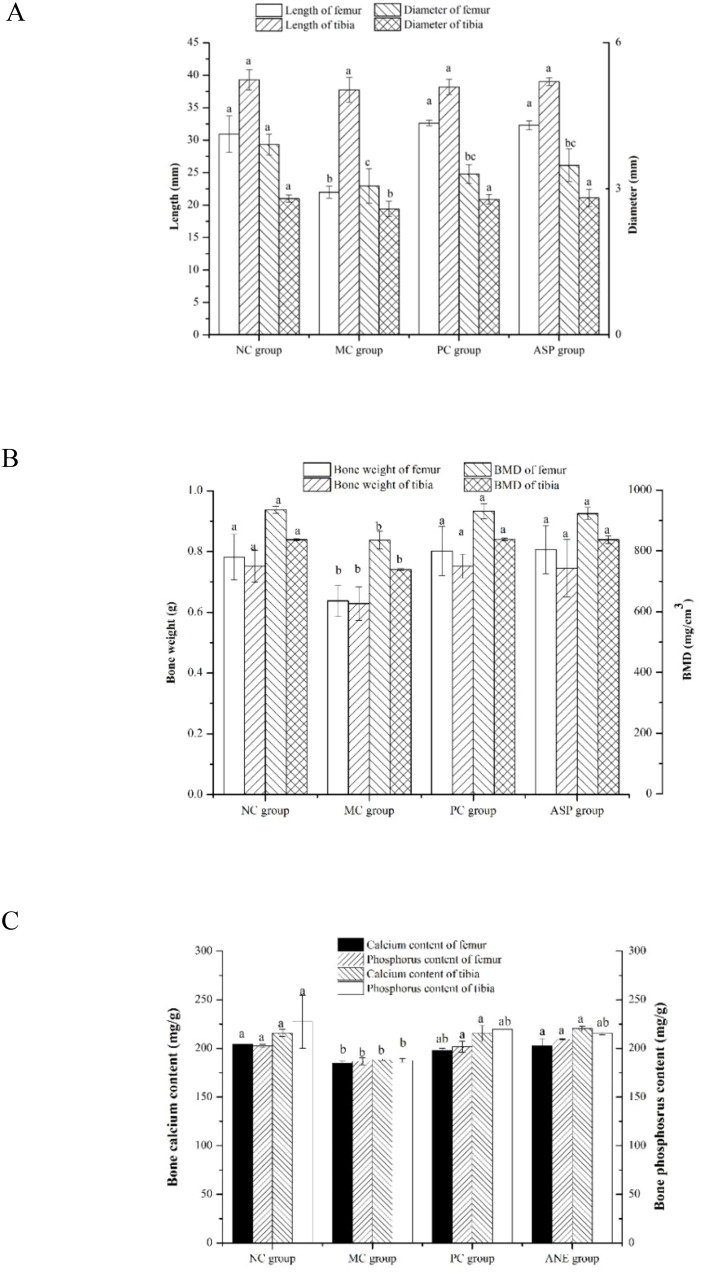
Effects of ASP on bone histomorphometry parameters in retinoic acid-induced osteoporosis rats. **(A)** Effects of ASP on length and diameter of femur and tibia in retinoic acid-induced osteoporosis rats. **(B)** Effects of ASP on bone weight and BMD of femur and tibia in retinoic acid-induced osteoporosis rats. **(C)** Effects of ASP on bone calcium and phosphorous contents in retinoic acid-induced osteoporosis rats.

Bone histomorphology parameters can intuitionistically reflect the effects of the tested sample on bone loss in osteoporotic animals. We determined the bone length, bone diameter, bone weight and BMD of the rats (**Figure 6**). Femur length of the MC group was significantly shorter than those of all other groups (*p<*0.05, **Figure 6A**). However, there were no significant differences in the lengths of tibias in all the groups (*p*>0.05). The diameters of femurs and tibias in the MC group were significantly lower than those in the NC group (*p*<0.05). ASP and the positive control sample could significantly improve the tibia diameter of mice (*p*<0.05). Additionally, there was no significant difference of the diameter of femurs among the MC, PC and ASP group (*p*>0.05). The bone weights of femurs and tibias in the MC group were significantly smaller compared with all other groups (*p<*0.05, **Figure 6B**). No significant differences were observed in the weights of femurs and tibias among the NC, PC and ASP group (*p*>0.05). The variation trends of BMD value in femurs and tibias confirmed to the differences of bone weights among the four groups.

Bone consists of the mineralized and nonmineralized (osteoid) components of the cortical and cancellous regions (29). Calcium and phosphorus are the main components of bone minerals and important factors in the diagnosis of osteoporosis. The retinoic acid-induced bone calcium and phosphorus contents were also determined (**Figure 6C**). The levels of the two essential elements in NC group were significantly lower than those in the MC group (*p<*0.05). The treatment of ASP could significantly enhance the value of the two parameters in femurs and tibias (*p<*0.05) except that of tibia phosphorus (*p*>0.05).

#### 3.2.3 The effects of ASP on bone histopathology

**Figure 7:**
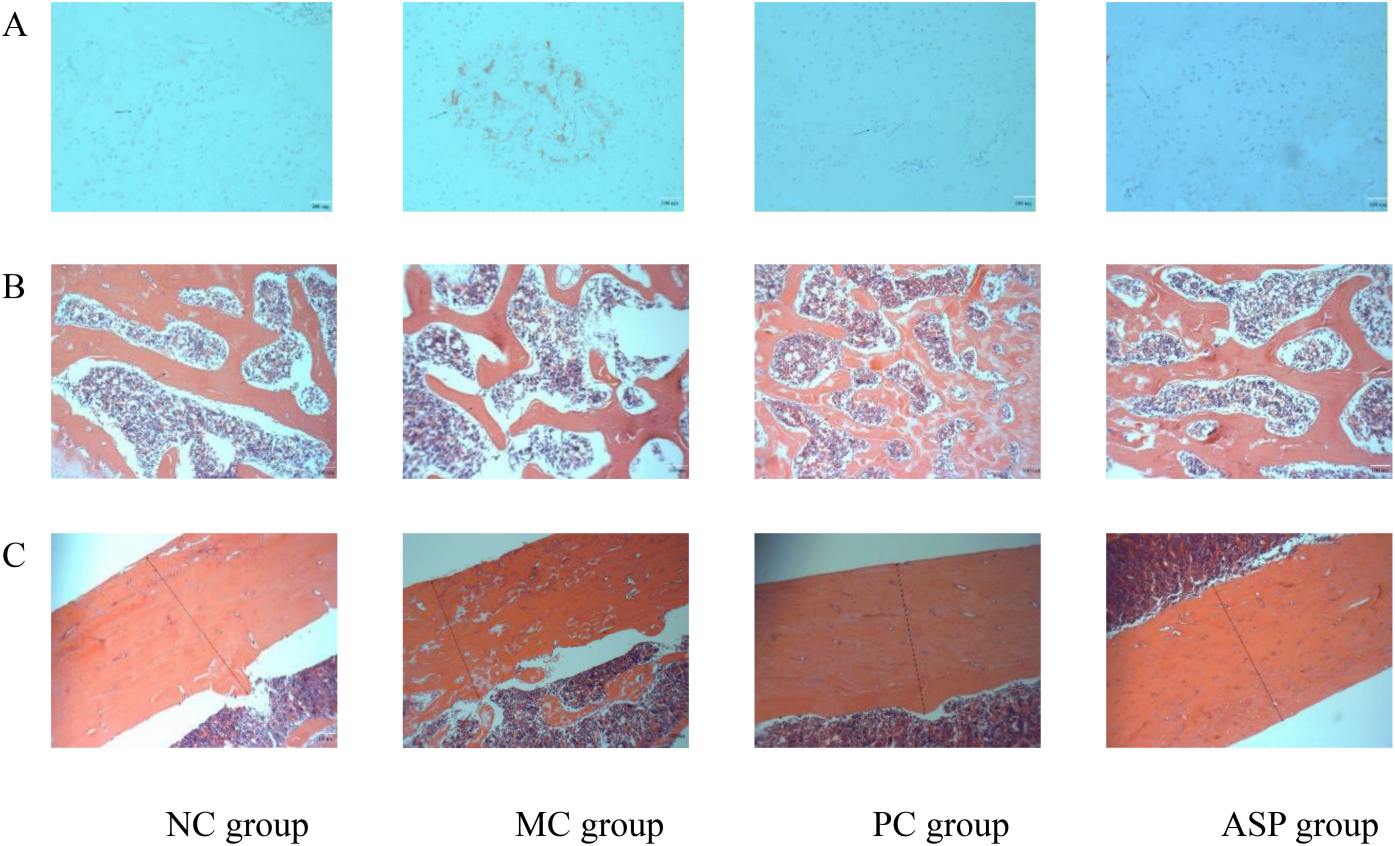
Effects of ASP on bone histopathology of retinoic acid-induced osteoporosis rats. **(A)** Effects of ASP on osteoclastogenesis in left tibias of rats (magnification of 100×). **(B)** Effects of ASP on trabecular microarchitectures of the left femur of rats (magnification 400×). **(C)** Effects of ASP on cortical microarchitectures of the left femur of rats (magnification 400×).

Bone health is maintained by the balanced activities of osteoblasts and osteoclasts. In the TRAP staining assay, cytoplasm of OCs will be stained claret-red and the nucleus will be in blue, which makes it effective to estimate the OC numbers and activity. The TRAP staining results of OCs in rat femurs from different groups are shown in **Figure 7**. In OP rats, the claret-red regions and number of blue nuclei were much more than those of NC group, which indicated that RA-administration induced the overactive of OCs. Compared with the MC group, ASP treatment decreased the area of claret-red regions and the amount of blue nucleus (**Figure 7A**) although there was no significant improvement at serum TRAP activity. The H&E staining of bone sections can visually exhibit the distribution of the trabecular distribution and cortical bone thickness (CBT). **Figure 7B** exhibited the architecture views of the right femurs from the experimental rats. The amount of trabecular bone decreased and the bone structure became sparse in OP, and this damage was obviously renovated in the ASP and positive control groups. In **Figure 7C**, the CBT was distinctly increased by ASP and alendronate sodium, compared with that of the MC group. In summary, ASP treatment could regulate the retinoic acid-induced abnormal changes of the trabecular bone in rats.

#### 3.2.4 The effects of ASP on microstructure of bone

To quantify the regulating effects of ASP on bone loss in RA-induced osteoporotic rats, bone microstructure was scanned according to mirco-CT views and the value of BMD, CBT, CAR and TAR were also calculated.

**Figure 8:**
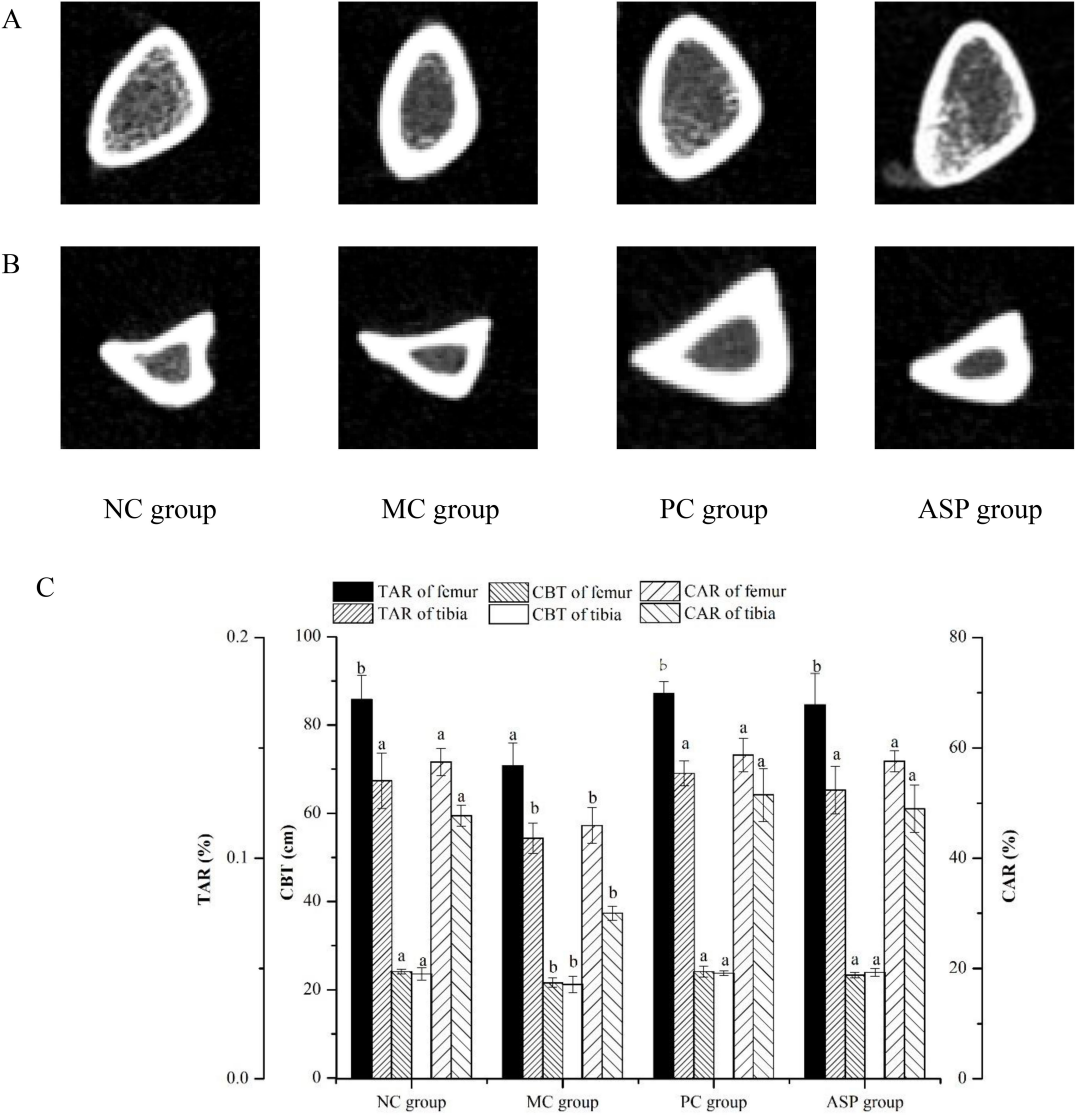
Effects of ASP on micro-CT views of femur and tibia of retinoic acid-induced osteoporosis rats. **(A)** Micro-CT views of left femur. **(B)** Micro-CT views of right tibia. **(C)** TAR, CBT and CAR calculated based on the mirco-CT views.

**Figure 8A** listed the micro-CT views of the femoral head specimens from rats with different treatments. At the end of the *in vivo* experiment, the RA-induction resulted in a serious decrease of the trabecular number, a more increscent space between trabeculars and a looser internal arrangement in the MC group. ASP gavage notably improved those appearances. The cortical bone thickness of tibias in MC group is obviously thinner, while there is no significant change in the cortical bone thickness between PC and ASP group, compared with that of the NC group (**Figure 8B**).

The value of TAR, CBT and CAR value of left femurs were markedly lowered in the MC group (*p*<0.05) and significantly improved by the ASP treatment (*p*<0.05). Additionally, there were no significant differences of TAR, CBT and CAR between the ASP group and the PC group, which meant that ASP could be a potential alternative in OP therapy. All those results indicated that ASP could ameliorate the bone damage induced by retinoic acid in rats.

**Figure 9:**
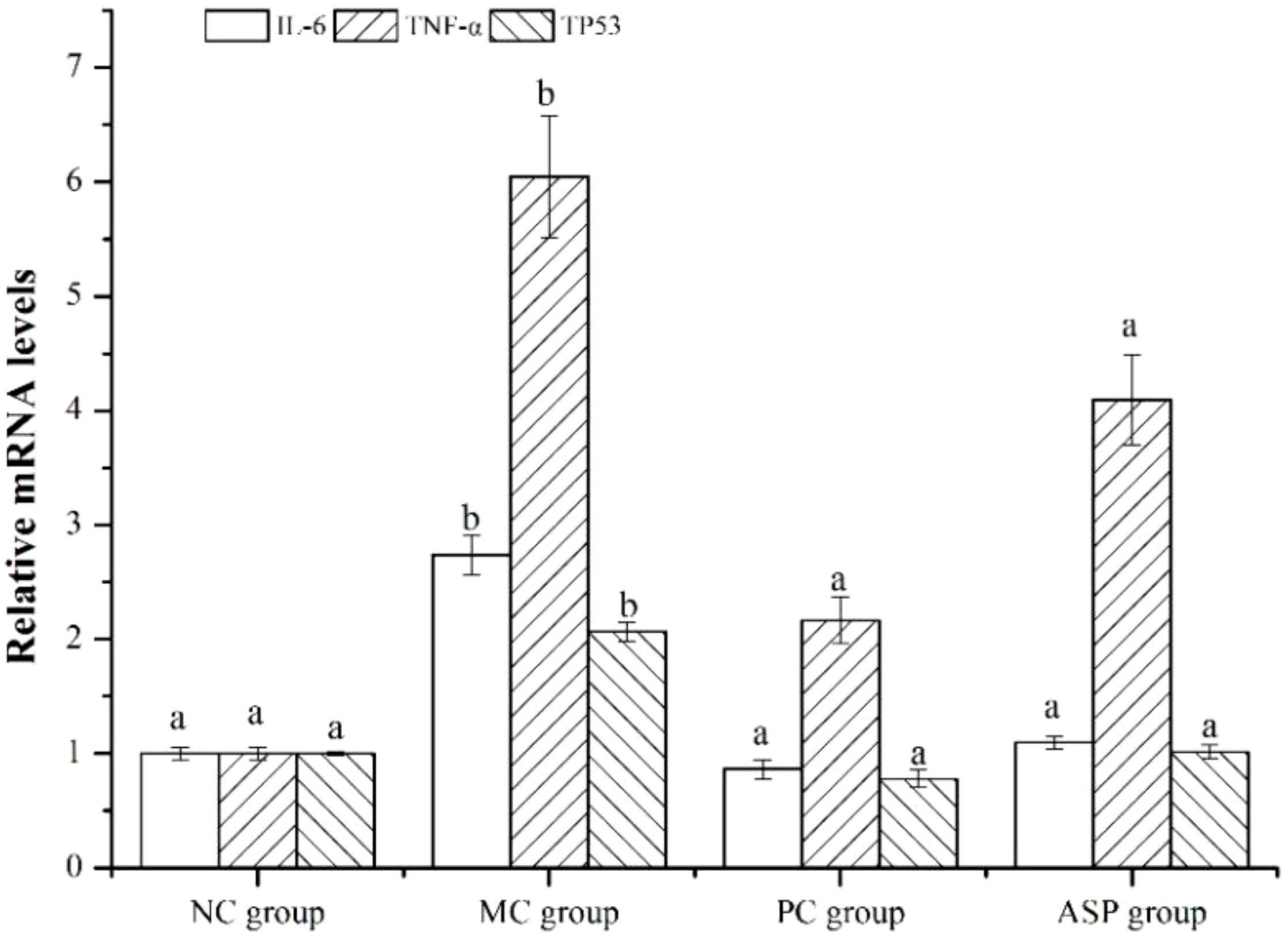
Effects of ASP on genes of right femur in in retinoic acid-induced osteoporosis rats.

To verify the results of network pharmacology method, RT-qPCR was employed to determine the expression of the three screened hub genes in left femurs of the rats. The results showed that the expression of these three genes (TNF-α, IL-6 and TP53) was significantly downregulated by the ASP treatment in the bone of RA-induced osteoporotic rats (**Figure 9**), which primarily confirmed the predicted results by network pharmacology.

## 4 Discussion

Medicinal plants are world widely used alone or as a supplement in traditional medicine. Phenols are second only of carbohydrates in abundance in medical plants and they play an irreplaceable role in their therapy for various human diseases, such as ischemic stroke (30), type 2 diabetes, hyperlipidemia, atherosclerosis and gout (31). Thus, phenolic compounds have gained worldwide attention and recognition in preventing health disorders. In our previous study, areca nut seed was rich in phenols, and its phenolic extraction displayed well as an antioxidant, hypoglycemic and antihypertension (2).

As a metabolic bone disease, OP is a severe public health concern which afflicts more than 200 million people worldwide, especially those over 50 years old (32). OP has been defined as one of the silent disease in the 21st century (33) and becomes a public health risk due to its severity, chronicity and progression. More and more phenolic extractions and compounds exhibited their high therapeutic effects on OP with high safety profile (34). The phenols from areca nut seed was reported that it could relieve osteoporosis through regulating the expression of osteoprotegerin (OPG), RANKL, LDL-receptor relater protein 5 (LRP5), bone morphogenetic protein-2 (BMP2) and β-catenin related to the Wnt/β-catenin pathway in VOX-induced OP rats (8). However, there are various acknowledged molecular signaling pathways contributing to OP occurrence and development, including RANK/RANKL/OPG, Wnt/β-catenin, and NF-κB signaling pathway. Moreover, due to the complexity and diversity of its component targets in the extractions of medical plants, it is difficult to comprehensively elucidate its mechanism of action in the treatment of diseases (2). Except those classical signals playing important roles in OP, are there many other vital signal transducting cascades involved in this disease and can be the therapeutic targets? Therefore, this study aimed to explore potential targets on OP of the phenols from areca nut seed with network pharmacology and verify the calculation results in retinoic acid induced OP.

The network pharmacology analysis screened the potential targets, active compounds and action signaling pathways of ASP in treating osteoporotic rats. According to molecular docking calculation, the top three core targets (TNF-α, IL-6 and TP53) and the top three active phenolic compounds (kaempferol, isorhamnetin and acacetin) could combine well with each other. In accordance, we also obtained those pathways in ASP treating OP. The results of animal experiment indicated that ASP had a notable efficacy on the RA-induced OP. Compared with the negative model group, ASP therapy improved serum biochemical indexes, bone parameters and bone microstructure. The gene expression of TNF-α, IL-6 and TP53 in tibia were exactly altered after ASP treatment in the OP mice.

The relationship between primary OP and inflammation response has been studied for decades. It was usually accepted that occurrence and development of OP are closely related to a series inflammatory cytokines, presented in infectious, autoimmune, and hematological disorders, which can stimulate osteoclastogenesis, leading to osteoporosis (35). IL-6 and TNF-α were active in inflammation and immunity, play a role in physiologic and pathologic resorption of bone. IL-6 stimulates osteoclast formation and promotes bone resorption in conditions like estrogen deficiency and in severe skeletal diseases (36). The activities of ALP and TRAP, which were the marker enzymes of osteoblast and osteoclast, respectively, were regulated to the normal level in the ASP group. In correspondence, the results of TRAP staining of the left tibia indicated that the formation of osteoclast was indeed improved by the treatment of ASP. The genes expression of IL-6 was down regulated by ASP in the left femur at the end of the experiment. The improvement of OCN content may be also related to the inflammatory modulation of ASP as IL-6 in bone could directly bind to osteoclast to promote bone resorption and with decarboxylation and activation of OCN as a result. Thus, some IL-6 inhibitors, such as tocilizumab, have been developed to reducing bone loss in rheumatoid arthritis (37). TNF-α is classified as a pro-osteoclastogenic cytokine as it has been improved to be related to the increase of osteoblast apoptosis and promotion of osteoclastogenesis *in vitro* and *in vivo* (38). TNF-α can also directly induce osteoclast precursors to express Fos, which produces IL-1β by interacting with bone matrix proteins and inducing osteoclast differentiation autocrine. The results in this study indicated that TNF-α gene expression in bone issue of the RA-induced osteoporotic rats was also decreased in ASP group. Correspondingly, we had demonstrated that the ASP exhibited its anti-inflammatory effect according to significant inhibition on the secretion of TNF-α and IL-6 in lipopolysaccharide (LPS) treated mouse macrophages (39). For another, it had been proved that ASP could ameliorate osteoporosis by altering gut microbiome via microbial protein lysozyme and the immune system in estrogen-deficient rats. Gut microbiota (GM) plays an important role in bone metabolism and the pathogenesis of OP and bacterial extracellular vesicles (BEVs) can provide new solutions to OP treatment (40). Thus, we deduced that the inhibition on OP of ASP was mainly related to its inflammatory effect.

The results of our study indicated that bone parameters related to bone quality including the diameter, wet weight and BMD were ameliorated. The analysis of micro-CT photo exhibited the improved CBT, CAR and TAR in the rats of ASP group. Currently, TP53 becomes an emerging target in OP therapy as it has demonstrated its regulation effects on bone mass *in vivo* (41) and effects on the osteogenic differentiation ability in cell models (42, 43). The results of qRT-PCR and molecular docking indicated that ASP could inhibit gene expression and the active flavonoids could bond with TP53 protein. Therefore, we deduced that ASP treated OP induced by RA in rats by adjusting TP53 signal transduction. TP53 was a transcription factor and induced the expression of downstream mRNAs to inhibit osteogenesis in bone marrow mesenchymal stem cells. What was more, in the IL-17 pathway signal cascade, Interleukin 17A (IL-17A) induced the upregulation of TP53, which was involved in inflammation, which could in directly influence the bone metabolic balance?

There have been various studies about the bone-protecting properties of phenolic compounds and their containing plant. The anti-OP capacities of phenols were mainly contributed to inhibiting oxidative stress, exerting antibacterial effects, and regulating inflammatory response. Various studies have demonstrated that the anti-inflammatory effect of polyphenols can potentially against OP (44). In this study, the network pharmacology analysis screened kaempferol, isorhamnetin and acacetin as three top active compounds in OP therapy of ASP. Kaempferol, a natural flavonol abundant in many herbal medicines, has shown its protective effects against bone loss *in vivo* and *in vitro* (45, 46). This compound played its therapy on osteoporosis primarily through inhibiting the secretion of inflammatory cytokines (IL-6, IL-1β and TNF-α) and targeting the inflammation signaling pathways such as activating MAPKs cascades and regulate the NF-κB signaling pathway (44), which are downstream signaling transduction of inflammation. Kaempferol was also verified as one the key active compounds in qianggu Capsule in OP treatment, with IL-6 and TNF-α as two of the top ten ranked hub genes (44). Isorhamnetin is one of the most important active flavonols in many medical herbs and it displays anti-osteoporosis properties both in cell and animal models. It can regulate the RNAKL/RNAK/OPG signaling pathway in ovariectomy SD rats and inhibit NF-κB pathway and decrease of mRNA expression of characteristic genes in RANKL-induced osteoclast. In the relief effect of bone loss in osteoporotic rats of *Sambucus williamsii* Hance var. *miquelii*, kaempferol and isorhamnetin have been validated as two of the key active constituents though the results weren’t proved in the animal experiment (47). As for acacetin, it is a flavonol and known for its anti-inflammatory capacities. Acacetin has a unique mechanism against diabetic osteoporosis by targeting the RANK/RANKL/OPG signaling pathway. IL-6 and TNF-α are two downstream signals in the IL-17 pathway and can be activated by IL-17 in macrophages. All of the information above confirmed our speculation that ASP alleviated bone loss in osteoporotic rats by regulating the IL-17 pathway to inhibit the expression of IL-6 and TNF-α. To enhance water solubility and targeting specificity, phenolic compounds with anti-OP effects can be progressively nanocrystallized to apply in reversal of systemic bone loss in OP (48).

The potential mechanisms of ASP accommodating RA-induced OP were predicted by GO and KEGG analysis. It figures that the more targets enriched in one pathway, the more important this pathway was in the treatment of drugs on diseases. As the results indicated, pathways in cancers, lipid and atherosclerosis, and IL-17 signaling pathway showing a high correlation with ASP against RA-induced OP. IL-17 signaling pathway plays a highly versatile role in vital processes including host immune defenses, tissue repair, inflammatory disease pathogenesis, and cancer progression (49). The pathway could induce upregulation of proinflammatory cytokines, antimicrobial peptides and neutrophil-recruiting chemokines (IL-1β, IL-6, and TNF-α) in human and mice (50). On the other hand, activation of IL-17 pathway can increase the expression of many signaling molecules associated with bone metabolism, such as TNF receptor associated factor 6 (TRAF6), which in turn promote the proliferation and differentiation of osteoclasts. As a result, two nuclear factor of activated T cells cytoplasmic 1 (NFATc1) and proto-oncogene Fos (an sub unit of activator protein 1), tow transcription factors in osteoclast formation, will be activated to accelerate osteoclast characteristic gene expression, leading osteoclast-mediated bone resorption (51). Therein, TRAF6 is an important molecular bridge in many physiological processes, including adaptive immunity, innate immunity, and bone metabolism (52). So, our further study will focus on IL-17 signal pathway in ASP against OP and improvement of water solubility and targeting specificity of ASP. On the other aspect, to understand the effect of ASP on human OP, we are expecting to firstly launch a work on epidemiological investigation of the relationship between arecanut chewing and OP morbidity statistics, intending to cooperate with other professional organizations.

## 5 Conclusions

Network pharmacology screened the potential targets and mechanisms of phenolic extraction of arecanut seed in anti-OP. ASP could inhibit bone loss in osteoporosis by increasing serum and bone mineral contents, decreasing osteoclast activity and aggrandizing osteoblast activity. And as a result, bone mineral density, cortical bone thickness, area ratio of bone cortex and area ratio of bone in osteoporotic rats were finally improved. These results suggested that ASP had positive effects on RA-induced osteoporosis by regulating the expression of the core genes predicted by network pharmacology. However, much more studies are still needed to confirm the mechanisms of ASP acting in anti-OP.

## Data Availability

The raw data supporting the conclusions of this article will be made available by the authors, without undue reservation.
