# Supplementary material for: Protective effects of arecanut seed phenols in retinoic acid induced osteoporosis and the potential mechanisms explored by network pharmacology
Source: Front Endocrinol (Lausanne). 2024 Oct 10;15:1472146. doi: 10.3389/fendo.2024.1472146 (PMC11499182; doi:10.3389/fendo.2024.1472146)
Supplement: Supplementary file 1 [file DataSheet1.docx]

| Table S1 Primers used for RT-qPCR | | |
| --- | --- | --- |
| Gene |  | **Primer sequence, 5'-3'** |
| IL-6 | **Forward** | 5′ -ACAAGTCCGGAGAGGAGACT-3′ |
|  | **Reverse** | 5′-TTGCCATTGCACAACTCTTTTC-3′ |
| TNF-α | **Forward** | 5′-GTGATCGGTCCCAACAAGGA -3′ |
|  | **Reverse** | 5′-CGCTTGGTGGTTTGCTACG-3′ |
| TP53 | **Forward** | 5′-GTGATCGGTCCCAACAAGGA-3′ |
|  | **Reverse** | 5′-CGCTTGGTGGTTTGCTACG-3′ |
| β-actin | **Forward** | 5′-CCGCGAGTACAACCTTCTTGC-3′ |
|  | **Reverse** | 5′-TATCGTCATCCATGGCGAACTGG-3′ |

| Table S2 Information on active ingredients of ASP | | | |
| --- | --- | --- | --- |
| Molecule ID | Molecule Name | OB (%) | DL |
| MOL010489 | Resivit | 30.84 | 0.27 |
| MOL000004 | Procyanidin B1 | 67.87 | 0.65 |
| MOL000073 | ent-Epicatechin | 48.95 | 0.24 |
| MOL000492 | Cianidanol | 54.82 | 0.24 |
| MOL005828 | Nobiletin | 61.66 | 0.51 |
| MOL000354 | Isorhamnetin | 49.6 | 0.30 |
| MOL005190 | Eriodictyol | 71.79 | 0.24 |
| MOL004576 | taxifolin | 57.84 | 0.27 |
| MOL000098 | Quercetin | 46.43 | 0.27 |
| MOL001803 | Sinensetin | 50.55 | 0.44 |
| MOL001689 | Acacetin | 34.97 | 0.24 |
| MOL002714 | Baicalein | 33.51 | 0.18 |
| MOL002881 | Diosmetin | 31.13 | 0.27 |
| MOL002563 | Galangin | 45.55 | 0.20 |
| MOL005573 | Genkwanin | 37.13 | 0.23 |
| MOL004908 | Glabridin | 53.24 | 0.46 |
| MOL008400 | Glycitein | 50.47 | 0.23 |
| MOL002341 | Hesperetin | 70.31 | 0.27 |
| MOL005530 | Hydroxygenkwanin | 36.46 | 0.27 |
| MOL004425 | Icariin | 41.58 | 0.61 |
| MOL004425 | kaempferide | 73.41 | 0.27 |
| MOL000422 | Kaempferol | 41.88 | 0.24 |
| MOL000006 | Luteolin | 36.16 | 0.24 |
| MOL000230 | Pinocembrin | 57.56 | 0.20 |
| MOL004648 | Troxerutin | 31.59 | 0.28 |

| Table S3. Key node of the compound-target network and table of its topological features. | | | |
| --- | --- | --- | --- |
| Molecule name | Degree | Betweenness Centrality | Closeness Centrality |
| Kaempferol | 54 | 0.164917 | 0.473016 |
| Isorhamnetin | 47 | 0.055224 | 0.452888 |
| Acacetin | 47 | 0.039549 | 0.452888 |
| Luteolin | 45 | 0.100235 | 0.447447 |
| Glabridin | 45 | 0.049765 | 0.447447 |

| Table S4. Annotation of the top 10 KEGG pathways. | | | |
| --- | --- | --- | --- |
| GOID | Description | Hits | *p* value |
| hsa05200 | Pathways in cancer | AR, CCND1, BMP4, BRAF, CASP3, CASP7, CASP9, CCNA2, CDK2, CSF1R, NQO1, EDNRA, EGFR, ESR1, ESR2, GSK3B, GSTM1, HDAC1, HMOX1, IFNG, IGF2, IKBKB, IL2, IL4, IL5, IL6, MMP2, MMP9, NFKBIA, NOS2, PDGFRB, PIK3CA, PPARD, PPARG, MAPK1, MAPK3, MAPK8, MAP2K1, PTGS2, RARA, RARB, RXRA, STAT3, STAT6, TP53, WNT3A | 6.48E-49 |
| hsa05417 | Lipid and atherosclerosis | CASP3, CASP7, CASP9, CD40LG, MAPK14, GSK3B, ICAM1, IKBKB, IL6, LDLR, MMP3, MMP9, NFKBIA, NOS3, PIK3CA, PPARG, MAPK1, MAPK3, MAPK8, RXRA, SELE, STAT3, TNF, TP53, VCAM1, NCF1 | 3.89E-31 |
| hsa04657 | IL-17 signaling pathway | CASP3, MAPK14, GSK3B, IFNG, IKBKB, IL4, IL5, IL6, MMP3, MMP9, MMP13, NFKBIA, MAPK1, MAPK3, MAPK8, PTGS2, TNF, FOSL1 | 1.47E-25 |
| hsa05215 | Prostate cancer | AR, CCND1, BRAF, CASP9, CDK2, EGFR, GSK3B, IKBKB, MMP3, MMP9, NFKBIA, PDGFRB, PIK3CA, PLAU, MAPK1, MAPK3, MAP2K1, TP53 | 2.71E-25 |
| hsa05207 | Chemical carcinogenesis - receptor activation | ADRB1, ADRB2, ADRB3, AHR, AR, CCND1, CYP1A2, EGFR, ESR1, ESR2, GSTM1, PGR, PIK3CA, PPARA, MAPK1, MAPK3, MAP2K1, RPS6KA3, RXRA, STAT3, NR1I3, UGT1A1 | 6.53E-25 |
| hsa05160 | Hepatitis C | CCND1, BRAF, CASP3, CASP9, CDK2, EGFR, GSK3B, IFNG, IKBKB, LDLR, NFKBIA, PIK3CA, PPARA, MAPK1, MAPK3, MAP2K1, RXRA, STAT3, TNF, TP53 | 1.72E-24 |
| hsa05418 | Fluid shear stress and atherosclerosis | ACVR1, BMP4, MAPK14, NQO1, GSTM1, HMOX1, ICAM1, IFNG, IKBKB, MMP2, MMP9, NOS3, PIK3CA, MAPK8, SELE, TNF, TP53, VCAM1, NCF1 | 5.74E-24 |
| hsa04668 | TNF signaling pathway | CASP3, CASP7, MAPK14, ICAM1, IKBKB, IL6, MMP3, MMP9, NFKBIA, PIK3CA, MAPK1, MAPK3, MAPK8, MAP2K1, PTGS2, SELE, TNF, VCAM1 | 6.12E-24 |
| hsa05161 | Hepatitis B | BRAF, CASP3, CASP9, CCNA2, CDK2, MAPK14, IKBKB, IL6, MMP9, NFKBIA, PIK3CA, MAPK1, MAPK3, MAPK8, MAP2K1, STAT3, STAT6, TNF, TP53 | 1.18E-22 |
| hsa05145 | Toxoplasmosis | ALOX5, CASP3, CASP9, CD40LG, CCR5, MAPK14, IFNG, IKBKB, IL10, LDLR, NFKBIA, NOS2, MAPK1, MAPK3, MAPK8, STAT3, TNF | 2.02E-22 |

| 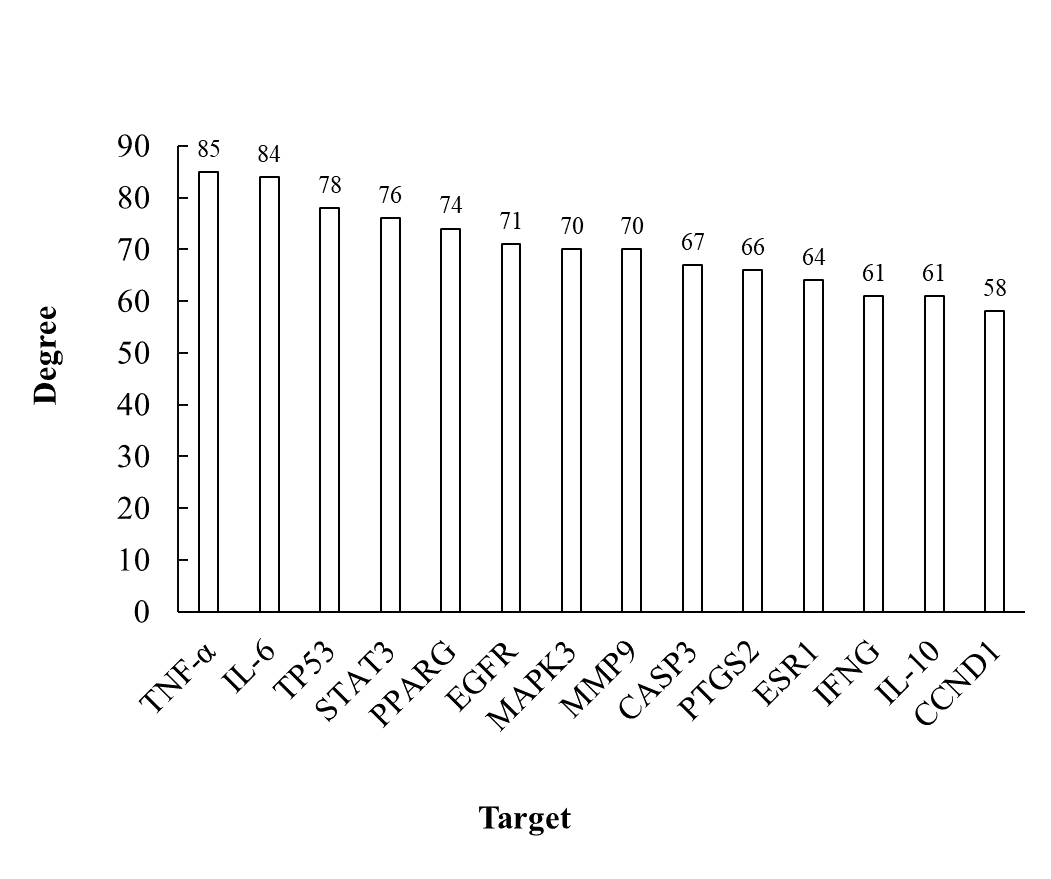 |
| --- |
| Figure S1. Core targets of ASP in the treatment of OP (top 15). |
